# An unusual case of thoracic empyema caused by *Granulicatella elegans* (nutritionally variant streptococci) in a patient with pulmonary tuberculosis and human immunodeficiency virus infection

**DOI:** 10.1099/jmmcr.0.005058

**Published:** 2016-10-27

**Authors:** Nomonde R. Mvelase, Kanitha Marajh, Olga Hattingh, Koleka P. Mlisana

**Affiliations:** ^1^​ Department of Medical Microbiology, University of Kwazulu-Natal, 800 Bellair Road, Mayville, Durban, South Africa; ^2^​ National Health Laboratory Service, 1 Modderfontein Road, Sandringham, Johannesburg, South Africa; ^3^​ Respiratory and Meningeal Pathogens Reference Unit, National Institute for Communicable Diseases, a division of the National Health Laboratory Service, 1 Modderfontein Road, Sandringham, Johannesburg, South Africa

**Keywords:** Thoracic empyema, *Granulicatella elegans*, pulmonary tuberculosis, Penicillin

## Abstract

**Introduction:**

Nutritionally variant streptococci (NVS) are an infrequent cause of human infection with *Granulicatella elegans* being the least encountered species in clinical specimens. The most common infection caused by NVS is infective endocarditis.

**Case Presentation:**

We report an unusual case of thoracic empyema due to *G. elegans *in a patient with pulmonary tuberculosis (TB) and human immunodeficiency virus infection. The patient responded favourably to drainage and penicillin.

**Conclusion:**

This case illustrates that even though TB is responsible for the majority of pleural effusions in this setting, other rare opportunistic bacteria may cause infection in susceptible patients. Therefore, microbiological investigations should be performed in all patients presenting with pleural effusion.

## Introduction

Nutritionally variant streptococci (NVS) were originally named as such because of their resemblance to streptococci on Gram stain smears and the requirement of pyridoxal hydrochloride (vitamin B_6_) or cystein for growth (Carey *et al.*, 1975; Frenkel & Hirsch, 1961; Ruoff, 1991). Following DNA–DNA hybridization studies, Bouvet *et al*. (1989) named* Streptococcus defectivus* and *Streptococcus adiacens* as two species of NVS. It was later discovered, using 16S rRNA sequencing, that these species were distinct from other streptococci, therefore they were assigned a new genus, *Abiotrophia* (Kawamura *et al.*, 1995). Subsequently three new species were described which included *Abiotrophia elegans, Abiotrophia balaenopterae* and *Abiotrophia para-adiacens* (Kanamoto *et al.*, 2000; Lawson *et al.*, 1999; Roggenkamp *et al.*, 1998). Further molecular studies separated NVS into two separate genera, with *S. defectives* remaining in the *Abiotrophia* genus while all the other species have been reassigned to the new genus, *Granulicatella *(Collins & Lawson, 2000).

NVS are facultatively anaerobic Gram positive cocci that form part of the normal flora of the upper respiratory tract, gastrointestinal tract and urogenital tract in humans (Ruoff, 1991; Sato *et al.*, 1999). They are an infrequent cause of infection, with the majority of cases presenting as infective endocarditis, accounting for 5 % of streptococcal endocarditis (Brouqui & Raoult, 2001; Roberts *et al.*, 1979). Although NVS have been implicated in pneumonia, to our knowledge only one case of thoracic empyema has been reported in the literature (Christensen & Facklam, 2001; Koh *et al.*, 2014; Liao *et al.*, 2004). Herein we report a case of empyema due to *Granulicatella elegans *in a patient with pulmonary tuberculosis (TB) and human immunodeficiency virus (HIV) infection.

## Case report

A 30-year-old female presented with a two week history of dyspnoea, productive cough and fatigue. Other complaints included loss of appetite and weight. She had been diagnosed with pulmonary TB and HIV infection two months prior to this presentation. Her CD4 count was 363 cells/mm^3^ and the viral load was 1737 copies/ml. She was not on anti-retroviral therapy. The TB diagnosis was initially based on clinical features as the Xpert MTB/RIF (Cepheid GeneXpert, Sunnyvale, Ca, USA) sputum results were negative. She was then started empirically on the standard first line TB treatment (rifampicin, isoniazid, pyrazinamide and ethambutol). However sputum culture (MGIT 960 system, BACTEC MGIT Becton Dickinson, USA) later confirmed drug susceptible *Mycobacterium tuberculosis* (MTB). There was no history of smoking or alcohol consumption.

Physical examination revealed that she was apyrexial with a temperature of 36.5 °C. She was in respiratory distress with pleural effusion involving the entire left lung which was confirmed on the chest X-ray. On examination of the cardiovascular system, she was pale, had a sinus tachycardia with a heart rate of 160 beats per minute and a blood pressure of 112/65 mmHg. There were no abnormal heart sounds or murmurs. Examination of the abdomen and the central nervous system was unremarkable.

Laboratory investigations showed severe anaemia with a haemoglobin of 5.4 g dL^−1^, a raised white cell count of 15.7×10^9^ L^−1^ and thrombocytosis of 958×10^6^ L^−1^. Urea and electrolytes were normal while liver function test results were as follows: total protein 90 g L^−1^, albumin 20 g L^−1^, total bilirubin 10 umol L^−1^, alanine transaminase 40 U L^−1^, aspartate transaminase 65 U L^−1^, alkaline phosphatise 228 U L^−1^ and gamma glutamyl transferase 88 U L^−1^. The blood cultures taken on the day of admission were negative after five days of incubation using the BacT/ALERT® automated system (bioMérieux, Marcy l'Etoile, France). The C-reactive protein and erythrocyte sedimentation rate were high at 217 mg L^−1^ and 105 mm h^−1^, respectively.

An intercostal drain was inserted into the left lung which initially drained 500 ml of pus, some of which was sent for laboratory analysis while the patient was treated empirically using ceftriaxone. First line antiretroviral treatment (tenofovir, lamivudine and efavirenz) was also initiated. No MTB was detected from the pleural fluid using Xpert MTB/RIF, unfortunately, mycobacterium culture of the fluid was not performed.

Laboratory analysis of the pleural fluid showed glucose of 0.1 mmol L^−1^, protein of 62 g L^−1^, while the lactate dehydrogenase was 20 799 U L^−1^. The Gram stain revealed many leukocytes with numerous Gram-positive cocci in pairs and short chains. The pus was cultured onto chocolate agar, 5 % horse blood agar, Mac-Conkey agar and colistin nalidixic acid agar (Diagnostic Media Products, National Health Laboratory Services, Sandringham South Africa). A pure heavy growth of small pin-point size, non-haemolytic colonies was observed on the blood plate after 48 h of incubation in CO_2 _with no growth on any of the other plates. The catalase and oxidase tests were negative.

The Satellite test was performed on both chocolate and 5 % horse blood agar using a streak of *Staphylococcus aureus* ATCC 25923. The organism demonstrated satellism on chocolate agar plate but normal growth was observed with no satellism on the blood plate. The VITEK 2 Gram-Positive Identification Card (bioMérieux SA, France) identified the organism as G* elegans *with 97 % probability identification index. This identification was later confirmed by the matrix-assisted laser desorption ionization – time of flight mass spectrometry (MALDI-TOF MS; VITEK® MS, bioMérieux)

Antimicrobial susceptibility testing was performed according to Clinical Laboratory Standards Institute (CLSI) guidelines for *Streptococcus* species, Viridans group, however testing was done on horse blood as isolate failed to grow on sheep blood. The penicillin Etest was susceptible at 0.023 µg mL^−1^. Repeated attempts on broth microdilution (Sensititre®, TREK Diagnostic Systems, USA) with cation adjusted Mueller-Hinton broth supplemented with 5 % lysed horse blood yielded no growth. The treatment was then changed to penicillin and the patient improved and was subsequently discharged after three weeks in hospital.

## Discussion

Among the NVS, the most common species that cause human infections is *G. adiacens*. *G.* *elegans *is rarely encountered in human specimens, probably because it was found to constitute the least amount of NVS isolated from the human mouth (Sato *et al.*, 1999). In the largest case series of NVS from human clinical specimens involving 97 patients, *G. elegans* constituted 3 % of infections (Christensen & Facklam, 2001). As with other NVS, *G. elegans* is most commonly recovered from blood cultures. Other NVS infections that have been reported in the literature include central nervous system infections, eye infections, bone and joint infections, abscesses, biliary tract infections, peritonitis and pneumonia. Liao *et al*. 2004 reported a case of thoracic empyema among 28 cases of NVS infections that occurred in Taiwan over a period of ten years. Although no specific clinical details were given for this case, the majority of patients in the case series had underlying diseases which included cardiac disease, malignancy, liver cirrhosis and diabetes mellitus.

Whilst a case of immunosuppression due to HIV has been reported as a risk factor for NVS in one case series, HIV is not a recognised predisposing factor for infection with NVS, probably due to the infrequent occurrence of these infections (Christensen & Facklam, 2001). This was the most likely predisposing factor in our patient. In addition, effects of TB infection on the lung immune defence may predispose to infection with other bacteria (Moore *et al.*, 2010). Dual pulmonary infection involving TB and other bacteria, particularly pneumococcus, has been reported in HIV infected patients from countries with high TB prevalence, causing diagnostic dilemmas for clinicians (Schleicher & Feldman, 2003). Given the high incidence of TB and HIV co-infection in our setting, TB was considered the most likely cause of the pleural effusion (WHO Global TB Report, 2014).

South Africa adopted Xpert as a first-line test for TB diagnosis following the World Health Organization recommendation in 2011 (WHO, 2013). Despite this, MTB culture is still considered the gold standard due to its superior sensitivity. HIV infected patients tend to have paucibacillary TB which can be missed by the Xpert (Boehme *et al.*, 2011; Mugusi *et al.*, 2006). Consequently, as shown in this case where Xpert was negative on the sputum while MTB culture was positive, MTB culture should be performed in cases of HIV and TB co-infection with Xpert negative results. Unfortunately since only Xpert was performed on the pleural fluid, dual pleural infection with both *G. elegans* and MTB could not be confirmed.

The fastidious nature of NVS as well as being slow growing can delay the isolation from clinical specimens. Special growth requirements have been shown by using media containing cysteine and pyridoxal hydrochloride or by satellite growth adjacent to *Staphylococcus aureus* (Reimer & Reller, 1981). It is generally accepted that NVS grow well on chocolate agar because it contains the required nutrients while satellitism on blood agar is required. (Koh *et al.*, 2014). However, contrary to this, our isolate grew well on 5 % horse blood agar while showing satellitism on the chocolate agar. A case of *G. elegans* that grew well on blood agar containing cystein but only grew poorly on chocolate agar was reported by Liao *et al.*, 2004. Similarly, a study done by Roggenkamp *et al.*, 1998 showed that unlike other NVS, growth of *G. elegans* was only supported by cystein and not by pyridoxal hydrochloride. Furthermore, a recent study by Koh *et al* (Koh *et al.*, 2014) demonstrated that discrepant satellitism can occur due to different kinds of nutrients found in blood agar media from different manufacturers.

Conventional biochemical identification (API-20 Strep, BioMéieux) of *G. elegans* is considered inadequate as it can lead to misidentification (Cargill *et al.*, 2012). Vitek 2 and MALDI-TOF (VIitek MS) have been used with promising results (Adam *et al.*, 2015; Christensen *et al.*, 2012). In a study done by, (Ratcliffe *et al.*, 2013) Vitek MS, was superior to Bruker MS and Vitek 2 for the identification of NVS. Molecular methods, in particular 16S rRNA gene sequencing, can be used to aid identification, especially where clinical specimens are culture negative (Breitkopf *et al.*, 2005; Casalta *et al.*, 2002).

Resistance to β-lactams has been reported; therefore susceptibility testing should be performed for all NVS isolates from infections that require treatment with antibiotics (Douglas *et al.*, 1994; Jorgensen & Hindler, 2007; Liao *et al.*, 2004; Zheng *et al.*, 2004). The recommended method for susceptibility testing is the broth microdilution method, although the E-test for penicillin showed comparable results (Douglas *et al.*, 1994; Jorgensen & Hindler, 2007). Our isolate was susceptible to penicillin by E-test.

The mainstay of treatment of empyema is drainage and antibiotics. Empiric therapy should be chosen based on the most likely pathogens and the duration of therapy depends on the clinical response. According to the American College of Chest Physicians classification of parapneumonic effusions, this patient would be category 4 effusion based on the fact that the fluid was pus (Light, 2006). Category 4 effusion is associated with a high risk of poor outcome and requires drainage in addition to appropriate antibiotics. Our patient responded favourably to three weeks of closed tube drainage and appropriate antimicrobials.

In conclusion, we report an unusual case of empyema due to *G. elegans* in a patient with pulmonary tuberculosis and underlying advanced HIV infection. This case illustrates that even though TB is responsible for the majority of pleural effusions in this setting, other rare opportunistic bacteria may cause infection in susceptible patients. Therefore, microbiological investigations should be performed in all patients presenting with pleural effusion.
